# Kevin N. Sheth, MD, receives the 2026 Bruce F. Scharschmidt and Peggy S. Crawford Translational Medicine Award

**DOI:** 10.1172/JCI209633

**Published:** 2026-07-15

The American Society for Clinical Investigation (ASCI) honors Kevin N. Sheth, MD (Figure 1), with the 2026 ASCI/Bruce F. Scharschmidt and Peggy S. Crawford Translational Medicine Award. Dr. Sheth is recognized for his transformative work deploying portable, low-field MRI systems allowing a safe, patient-centered bedside diagnosis. He is director of the Yale Center for Brain and Mind Health and vice chair for Clinical and Translational Research in the Departments of Neurology and Neurosurgery. Dr. Sheth was elected to the ASCI in 2020. Immediate Past President Dr. Anna Greka of the Broad Institute, Harvard Medical School, and Brigham & Women’s Hospital, interviewed Dr. Sheth at the ASCI/Association of American Physicians/American Physician-Scientists Association Joint Meeting in May 2026.

Anna Greka: Congratulations on receiving this award. Could you describe how you became interested in your research question and the journey for you and your team in coming to these discoveries and their impact on patients?

Kevin N. Sheth: I just want to say, on behalf of our research teams and our trainees, how grateful we were to receive this award, especially [one named for] a leader who has thought about translational medicine in a fundamental way and how it can be disseminated and applied in the real world. I’m a vascular and critical care neurologist and a physician-scientist at Yale. Our group’s work has increasingly centered on a single, broad question: How do we scale brain health and neurological care to populations that current biology and systems simply may not reach very well? From early on, I was always drawn to both the science and the idea that systems can be redesigned within medicine. I found neurology to be intellectually fascinating but also frustrating sometimes because neurological disease is devastating, it is common, and it is often detected too late. I became interested in things like stroke, both acute stroke treatment and prevention of things like stroke and dementia, because it sits at the intersection of emergency care, chronic disease, imaging, prevention, technology, and health systems. And for the project that is the subject of this award — the development and deployment of portable MRI systems — what really pushed us was that we were trying to develop through translational programs biological targets that were implicated in acute brain injury, brain swelling, and early forms of injury. We needed to develop pharmacodynamic markers, that is, brain imaging markers that would allow us to see whether our interventions were working where none existed. This was very challenging to do in critically ill patients. [Focusing on] imaging at high resolution at the bedside was a natural way to both develop the technology in an interdisciplinary [approach] and to facilitate the translational development of exciting biological discoveries.

AG: That’s remarkable and empowering, I would imagine, for the colleagues who also now have the opportunity to use this technology. Could you speak about the ease or difficulty with which you were able to take this from what was happening in your laboratory, within your own team, to something that can be used more widely?

KNS: It’s been an interesting journey, and like a lot of these things, as you know, it was certainly not a linear path. Multiple laboratories in this field have been working for decades on low-magnetic-field technology — the opposite of most MRI machines, which involve higher and higher magnetic fields. But that had been living in the lab. In order to get [the technology] translated, we faced the challenge of finding a funding source, because this technology sat at an intersection. Ultimately, we were able to develop an exciting academic-industry-NIH partnership that allowed us to put teams together that included physicists, engineers, and clinical physician-scientists like myself. The challenge then was to take a prototype that we were able to iterate off of and deploy it in a clinical setting. To be honest, at the very beginning, when we were thinking about the engineering, the science, the physics, the clinical use cases, we didn’t always think about things like form factor or what it would mean in a nursing workflow or how you would wheel the device through the hospital. And what about all the machines such as dialysis machines and EKGs that might provide electromagnetic interference? These were common, maybe mundane, things that became very relevant, and through a process of iteration, we were able to get there. I will end by saying that we had ideas about the disease context in which we were going to validate the use of this new tool or technology, but the registration data came during the COVID pandemic. That was a time in the early days in which patients could not easily undergo brain imaging for infection control reasons. It ended up being the use case that provided us the registration data.

AG: Where there’s difficulty, there’s opportunity. What kind of lessons have you learned along the way in your career that could be useful to a young neurologist or physician-scientist working in this space? What would your message be to some of our younger colleagues?

KNS: One [lesson] is that, as I mentioned, careers are often less linear than they appear from the outside. Sometimes people assume that successful scientists follow a clean upward trajectory, but most careers involve uncertainty, failed ideas, setbacks, and redirections. We sometimes enrich for people who are risk-averse in our environment, but I would say, Run toward risk. A second lesson is to choose important problems rather than just fashionable techniques. Technologies change rapidly, and the underlying human problems are often more durable. I’d also encourage trainees to cultivate breadth without losing depth and never think of yourself [as being at a particular] level. Sometimes, in a good way of progressive responsibility, our systems are designed to see one, do one, teach one. But at the same time, I have always been impressed by trainees who don’t think of themselves as being at a [particular] level and oftentimes come up with insights just as frequently as someone who’s been practicing or doing science for thirty or forty years. So just think that what you are doing is the most important thing in the world and that there is nothing you can’t do at any point in your career.

AG: Picking up a little bit on what you said earlier — that it took a collaboration between academia and federal funding and industry to make this a reality: It’s often the case that colleagues might think that forming partnerships with any of these different entities might be challenging or difficult. What were some of the lessons you learned, and would you encourage others to pursue these kinds of multidisciplinary approaches and multi-institutional relationships that can facilitate such discoveries?

KNS: I am still surprised to hear that there are [such] perceptions about working with generically different groups of partners. But if your approach, irrespective of your partner or your funding source, is one of integrity, rigorous science, and high-quality work that you disseminate openly and freely in a transparent way, then you can and you should partner with anyone who can push the science forward for big problems. The second thing is that different partners have different needs and pressures, and putting yourself in other people’s shoes and thinking about what those [needs and pressures] might be is important. I don’t think it’s intrinsically wrong to [consider] commercial pressures that a drug developer or technology may have. It [might not be] my personal interest, but understanding that it’s one piece of the puzzle can help align the opportunities, and it shows true respect for the partnership. We would hope that they would do the same for us [with regard to] our interests, for example.

The last thing — and I think this is true for different partners, including in the academic domain — none of us is naive, but at the same time, [you need to] be willing to give up credit, to be willing to share. We know how much pressure there is sometimes to be the contact PI or the first or last author. But I’ve found, in general, in working with teams, if everybody is going toward an important goal, you can think about how to grow the pie and then split it, as opposed to just splitting the existing pie. And I’ve found that when you give things up in the short run, it can turn out to be a bigger win in the long run most of the time. Doing it that way allows you to succeed, or it helps make it a little more likely, and it’s certainly a lot more fun.

AG: Clearly, you’ve made a tremendous amount of progress, but in which directions do you see this work moving forward?

KNS: We are so excited about this technology. I think developing new tools in science allows us to make new observations, which then sets the stage for a lot of hypotheses. We believe that’s what’s happening here. If you were to just look at the number of papers [published] or labs that were studying this technology five years ago versus now, you would see an exponential increase. So to be part of a [group] of people who are developing and advancing a new field is super exciting. To give you a couple of quick, concrete examples: Number one, what you get when you go to a low magnetic field, as you can imagine, is a tradeoff with image quality and resolution. But at the same time, today we can deploy techniques using image reconstruction and artificial intelligence that have been broadly applied to a lot of imaging fields and bring that to ours. That is going to continue to go forward. On the other hand, as a critical care and emergency neurologist, we validated a lot of this work in the acute inpatient setting. But I think the most exciting use is going to be out in the community. MRI was one of the most successful technologies of the twentieth century, but today it is not available to most of the people on the planet. Now that this [machine can go] on wheels, you can put it in outpatient clinics, in remote villages, or in the back of a van. Making access to this technology equitable and making new observations in the community — that’s a very exciting frontier.

AG: Are you aware of programs that are bringing this kind of technology to the Global South, for example, where there might not be as much MRI availability otherwise?

KNS: Absolutely. This year, the International Society for Magnetic Resonance in Medicine had their keynote meeting in South Africa. Also, the Bill & Melinda Gates Foundation has sponsored this work throughout Africa, where there are many scanners deployed. The first portable systems were just deployed in India, and they are now being used on virtually every continent. So we’re at the very early stages of this, and I’m very excited about it.

AG: I’m curious whether the tradeoff on resolution allows or does not allow us to think about tracers or other things that can be used to look at molecular markers in the brain, perhaps earlier in the progression of disease. What do you think about that combined with your technology?

KNS: Groups are asking those questions right now. Certainly, the idea is to use this technology in humans at earlier stages. There are people using this technology not just for earlier [assessment], but also for serial dynamic and spatial and temporal profiles to get serial images much more easily. Our group and others are using this [approach] in animal models in the preclinical setting. The development is at low magnetic field. As we speak, I know there are other groups on the academic and industry sides that are developing new contrast agents and new tracers that can be optimized for a low magnetic field. It’s still unknown; it’s not proven that these things are going to work, but I know there is a lot of investigation happening in that area.

AG: [Looking] back now with all the wisdom you have gained, how was it back when you were thinking about becoming a physician-scientist? How did you make certain decisions that brought you to where you are today? What words would you have for someone who is not yet a colleague?

KNS: First of all, be open and go where your heart goes. I will say that big initiatives — federal initiatives — make a difference. I became interested in neuroscience in the 1990s, which was a time when Congress and our federal government proclaimed it “the decade of the brain.” Because of that proclamation, it’s now ubiquitous, but I was at Johns Hopkins — one of the first neuroscience majors. I think that wouldn’t have happened if it hadn’t been for that government initiative that led to those programs that pulled me [into the field] in the first place. So keep your eyes open and look for these big things that are happening. Number two: Don’t go away from the crowd just for the sake of being different. On the other hand, I do remember that a lot of people were a little bit hesitant to do investigation in neuroscience or, certainly from a clinical perspective, to do things in neurology because, well, you could describe the problem, but what could you do?

I think anybody in neurology today remembers that era, and it has changed. The way I thought about it was that it’s such an important problem, it can’t stay that way forever. It has to change. So why not be a part of that change? Maybe we’ll push that change. I’ve had this funny thing — I’m sure you have as well — that when everybody tells you not to do something, not to go somewhere, it’s either a reason not to do something or it might be exactly where you should go. The diagnostics and therapeutics in the broad neuroscience landscape just continue to be at multiple dynamic inflection points. What an exciting time!

AG: Is there anything else you wanted to say in closing this conversation?

KNS: The main message is that the ASCI is such a wonderful, broad organization of physician-scientists and people who want to change the world. It has been a privilege for me to be a part of the ASCI and to be recognized by my peers; my team and I are so grateful.

*The interview has been edited for length and clarity*.

## Figures and Tables

**Figure 1:**
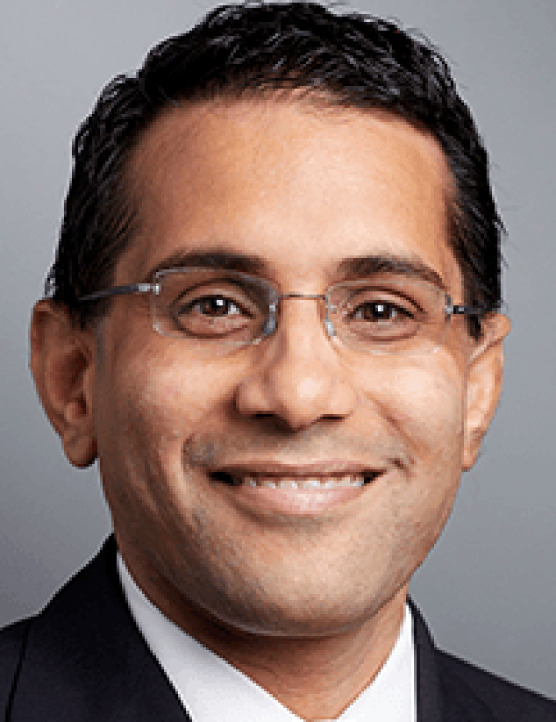
Kevin N. Sheth is the recipient of the 2026 Bruce F. Scharschmidt and Peggy S. Crawford Translational Medicine Award.

## Version 1 07/15/2026

Electronic publication

